# Chinese adolescents' coping tactics in a parent-adolescent conflict and their relationships with life satisfaction: the differences between coping with mother and father

**DOI:** 10.3389/fpsyg.2015.01572

**Published:** 2015-10-15

**Authors:** Hongyu Zhao, Yan Xu, Fang Wang, Jiang Jiang, Xiaohui Zhang, Xinrui Wang

**Affiliations:** School of Psychology, Beijing Normal UniversityBeijing, China

**Keywords:** adolescent, parent-adolescent conflict, conflict coping tactics, life satisfaction, adolescence

## Abstract

The present study examined the differences of conflict coping tactics in adolescents' grade and gender and parents' gender and explored the relationships among conflict frequency, conflict coping tactics, and life satisfaction. A total of 1874 Chinese students in grades 7, 8, 10, and 11 completed surveys on conflict frequency, coping tactics, and life satisfaction. The results obtained by MANOVA suggested that the adolescents' reported use of assertion and avoidance with either mothers or fathers increased from Grade 7 to Grade 8 and did not change from Grade 8 to Grade 11 in parent-adolescent conflicts. The results of paired sample *T*-tests indicated that adolescents used more conciliation in Grade 7, more conciliation and assertion in Grade 8, and more conciliation and less avoidance in Grade 10 and 11 to cope with mothers than with fathers in parent-adolescent conflicts. Boys used more conciliation and less avoidance, while girls used more conciliation, assertion and third-party intervention to cope with mothers than with fathers in parent-adolescent conflicts. The results of the hierarchical regression analysis indicated the significance of the primary effects of conflict frequency and coping tactics on life satisfaction. Specifically, conflict frequency negatively predicted life satisfaction. Conciliation positively and avoidance negatively predicted life satisfaction when adolescents coped with either mothers or fathers in parent-adolescent conflicts. Assertion negatively predicted life satisfaction when adolescents coped with fathers. The moderating effects of conflict coping tactics on the relationship between parent-adolescent conflict frequency and life satisfaction were not significant.

## Introduction

Parent-adolescent conflict is common and inevitable throughout adolescence (Smetana and Gaines, 1999). This conflict contributes to the formation and development of adolescent self-identity in the long term (Steinberg, 1990; Adams and Laursen, 2007). At the same time, parent-adolescent conflict has a negative impact on family harmony in the short term (Montemayor, 1983; Silverberg and Steinberg, 1987) and is positively related to internal and external problem behaviors (Smetana, 1996; Gil-Rivas et al., 2003), which may further influence adolescents' life satisfaction. It is a challenge for adolescents and parents to address various types of conflicts during adolescence. Among many factors, selecting proper ways to cope with parent-adolescent conflicts is more important than the conflict itself for adolescents' self-growth (Adams and Laursen, 2007). Bosma and Kunnen (2001) also proposed that a successful resolution of such conflicts can play favorably into identity development. On the other hand, the adolescents' use of conflict coping tactics were different in adolescents' grade and gender and parents' gender in parent-adolescent conflicts (Laursen et al., 1998; Van Doorn et al., 2011). According to gender theory (Thompson and Walker, 1989) and gender schema theory (Bem, 1985), parent gender and adolescent gender influence an adolescent's perception of parent-adolescent relationship and subsequently influence their coping tactics in parent-adolescent conflict. Therefore, it is of great importance to investigate the differences in adolescents' coping tactics according to their grade and gender and the parents' gender, and the effects of adolescents' coping tactics in the course of parent-adolescent conflicts on their self-growth.

Coping is generally defined as using a strategy for managing stress (McCubbin, 1979). Several scholars have identified conflict coping tactics as an important aspect of conflict (Shantz, 1987; Collins and Laursen, 1992) because these coping tactics were crucial for maintaining good relationships (Adams and Laursen, 2001). In real conflicts, there are three types of coping styles: actively resolving conflict (Ohbuchi et al., 1996), passively avoiding conflict (Ohbuchi and Takahashi, 1994) and seeking help from a third party (Pruitt and Carnevale, 1993). Based on these styles, Ohbuchi and Tedeschi (1997) defined four coping tactics: conciliation, assertion, third-party intervention and avoidance. Among these tactics, conciliation, and assertion are active and direct tactics. Conciliation is defined as an attempt to consolidate one's own and the other's goals and to alleviate the other's negative emotions. Assertion is defined as an attempt to assert one's request strongly and to demand that the other do something and to criticize or display anger at the other. Avoidance is a passive tactic and is defined as avoiding confrontations with others and stay self-control. Third-party intervention is defined as a tactic to seek help or advice from a third party to resolve conflicts.

Adolescents adopt different tactics to cope with parents in parent-adolescent conflicts according to adolescents' grade and gender and parents' gender. This viewpoint is supported indirectly by theories that emphasize the effects of parents' gender and adolescents' gender on the parent-child relationship. For example, gender schema theory emphasizes gender schematic processing based on socially prescribed gender schema for maleness and femaleness (Bem, 1985; Russell and Saebel, 1997). According to gender schema theory, gender (e.g., boy vs. girl, mother vs. father) may affect adolescents' behavioral reaction and coping style. Another theory is gender theory, which proposes that there are differences between mothers and fathers in family roles and behaviors, such as housework, childcare responsibilities, and other roles that parents play (West and Zimmerman, 1987; Thompson and Walker, 1989). These differences of interactions may strongly influence intimacy between adolescents and parents, and therefore affect adolescents' coping style in parent-adolescent conflicts. Similarly, Russell and Saebel (1997) hold the concept that the gender combination is important, boys' interactive behavior with their mothers will differ from behavior with their fathers, and it holds true for girls as well.

In addition to theoretical perspectives, several empirical studies also explored the differences in coping tactics according to adolescents' grade and gender and parents' gender. Previous studies indicate that coping tactics with fathers and mothers change with grades. A study on age differences in conflict resolution showed compromises with parents steadily increased from grades 8 to 10 (Owens et al., 2005). A longitudinal study of 314 early adolescents (13–14 years) showed that adolescents' reported use of positive problem solving with mothers increased, but did not change with fathers from early to middle adolescence, whereas the change of conflict engagement and avoidance were non-linear: adolescents' reported use of conflict engagement and avoidance with mothers first temporarily increased from 13 to 15 years and then decreased from 15 to 17 years but did not change with fathers (Van Doorn et al., 2011). In brief, positive coping tactics increase and negative coping tactics decrease with the advance of age for coping with mothers. Most of studies have examined grade differences of coping tactics, however, few studies considered parents gender differences. Therefore, the present study examined the differences in coping tactics with mothers and with fathers in each grade.

Previous studies indicated that coping tactics are different according to adolescents' gender. Osterman et al. (1997) studied gender differences of children and adolescents (8, 11, and 15 years of age) in Finland, Israel, Italy, and Poland. The results showed that girls used more constructive conflict resolution and third-party intervention than did boys. Many other studies also demonstrated that girl tended to seek more social support than did boys (Frydenberg and Lewis, 1991; Feldman et al., 1995). However, the study of Owens et al. (2005) of 591 adolescents (304 boys, 287 girls) found that girls reported higher levels of compromise, obliging, and avoidance than boys. It is obvious that the results of previous studies on gender differences are not consistent. Therefore, the present study examined adolescents' gender differences in coping tactics. In addition, according to the above-mentioned gender theories, we also explored whether adolescents' coping tactics with mothers and fathers were different for boys or girls.

Finally, the above-mentioned findings were conducted in Western cultures. It is well-known that there are large differences between Western culture and Chinese culture (individualism vs. collectivism culture). First, in the context of a collectivist culture, people prefer to use passive, collaborative, and avoidance tactics to maintain positive social relationships and to avoid or reduce conflicts (Juang et al., 2012). Thus, avoidance may increase with grade for Chinese adolescents. Second, the parent-adolescent relationship is dependent, rather than independent, in Chinese culture. In inter-generational communication, Chinese parents, as authority figures, possess the decision power on almost everything for their children (Cooper, 1999; Hwang, 2000). Moreover, child development in Chinese culture is known as a process of learning to forgo personal needs and desires to satisfy parental and societal expectations and maintain interdependence in the social network (Ho et al., 1991; Lam, 1997). Thus, assertion may decrease with grade for Chinese adolescents. Because of these differences in cultures, we assume that the changes of conflict coping tactics for Chinese adolescents may be different from those in Western culture.

A great deal of research indicated that parent-adolescent conflict frequency influences adolescents' self-development and family relationships and therefore influences their perception of life satisfaction. For example, previous studies indicated that parent-adolescent conflict frequency positively predicts negative consequences, including aggressive behavior (Smetana, 1996), depressed feelings (Gil-Rivas et al., 2003), and lower self-esteem (Deković, 1999). Parent-adolescent conflict frequency also affects family relationships (Cicognani and Zani, 2010), life quality and life satisfaction of family members (Silverberg and Steinberg, 1987). Moreover, adolescents' negative consequences, depression (Huebner et al., 2000), and lower self-esteem (Bradley and Corwyn, 2004) negatively relate to life satisfaction, and harmonious family relationships positively relate to life satisfaction (Leung and Leung, 1992; Henry, 1994). Therefore, we assume that parent-adolescent conflict frequency is negatively related to life satisfaction.

Several studies indicated that effective conflict tactics including problem-solving strategy, conciliation, improve self-esteem, and reduce depression and risky behavior (Tucker et al., 2003), whereas ineffective resolution approaches including conflict engagement, avoidance, increase adolescents' delinquency (Van Doorn et al., 2008). A clinical study from 61 adolescents (*M* = 15.9 years) also showed that maladaptive conflict resolution skills were positively associated with externalized behaviors, such as fighting, smoking, drinking, and marijuana use (Colsman and Wulfert, 2002). Thus, we hypothesize that effective conflict tactics (e.g., conciliation) will positively associate with life satisfaction, whereas ineffective resolution (e.g., avoidance) will negatively associate with life satisfaction.

Conflict coping tactics may moderate the relationship between conflict frequency and adolescents' life satisfaction. A few studies indirectly support the moderating roles of conflict coping tactics. For example, in a study (*N* = 131, 3 adolescents), five combined resolutions were distinguished using hierarchical cluster analysis, including withdraw, positive, negative, no, and very positive. The results indicated that as the frequency of conflict with their parents increased, adolescents who used the negative resolution style (the combined style of conflict engagement, withdrawal, and exit) reported more internalized problems, and adolescents who used withdraw resolution style (the combined type of withdrawal and lower positive problem-solving) reported more externalized problems (Branje et al., 2009). Furthermore, Laursen and Hafen (2010) depicted a moderating model of conflict types. Specifically, as conflict frequency increased, constructive conflict resolutions would produce more beneficial outcomes and less detrimental outcomes, whereas coercive conflicts would produce less beneficial outcomes and more detrimental outcomes. Therefore, in the present study, we assume that conflict coping tactics moderate the relation between parent-adolescent conflicts and adolescents' life satisfaction. In the mean time, we also investigate whether there is a moderating effect difference in coping with mothers and coping with fathers.

The present study, with a large sample of adolescents from mainland China, was conducted to explore the differences of Chinese adolescents' coping tactics in their grade and gender and parents' gender. Based on a review of previous studies on grade differences, we expect that among conflict coping tactics, the conciliation, and the avoidance increase with the increase of grade level; the assertion decreases with the increase of grade level; there is no relationship between third-party intervention and grade level, and finally, there are differences in coping tactics with mothers and with fathers in each grade.

According to gender schema theory (Bem, 1985) and gender theory (West and Zimmerman, 1987; Thompson and Walker, 1989), parent-adolescent relationship varies with adolescents' gender and parents' gender, which may influence interactions and intimacy between parent and adolescent, and then may have an effect on adolescents' coping tactics in parent-adolescent conflicts. Although many studies have examined the adolescents' gender differences in coping tactics, the conclusions from these studies are not consistent. We assume that there are the differences of coping tactics in adolescents' gender. Furthermore, few researchers have considered the differences in coping tactics with mothers and fathers for boys and girls. We assume that there are the differences in coping tactics with mothers and fathers for boys and girls.

The present study also examined the relationships among parent-adolescent conflict frequency, coping tactics, and adolescents' life satisfaction. Based a review of previous studies, we expect that conflict frequency is negatively related to life satisfaction. Among the coping tactics, we expect that the conciliation is positively related to life satisfaction, the assertion, and the avoidance are negatively related to life satisfaction, the third-party intervention is not related to life satisfaction. We also intend to detect the moderating effect of coping tactics on the relationship between conflict frequency and life satisfaction, and parents' gender difference in the moderating effects.

## Methods

### Participants

A total of 2072 adolescents, primarily ethnic Hans, were recruited from six public schools in Beijing, China. The final valid sample size was 1874 adolescents (817 boys and 1057 girls). The adolescents' age ranged from 13 to 19 years (*M* = 15.90, *SD* = 1.49). A total of 268 adolescents were in seventh grade (14.3%), 288 in eighth grade (15.4%), 795 in 10th grade (42.4%), and 523 in 11th grade (27.9%). A total of 198 participants were excluded as invalid. Among these participants, 101 did not complete the questionnaire, 51 had single parents, and 46 had responses of extreme value. The effective rate of the sample is 92.4%. In parent-adolescent conflicts, adolescents may adopt different coping tactics with mothers and fathers. Considering that adolescents may adopt different coping tactics with mothers and fathers in parent-adolescent conflicts, we had adolescents report their coping tactics with mothers and fathers in conflicts respectively in this study.

### Measures

#### Frequency of conflict

The frequency of conflict with parents was measured with the adolescent-parent conflict questionnaire (Fang et al., 2003). This questionnaire consists of three items: body conflicts (such as slapping, pushing, kicking), verbal conflicts (such as quarreling, arguing, mocking, scolding, shouting) and emotional confrontation (such as snubbing, being angry, being annoyed). On a 5-point Likert scale ranging from 1 (never) to 5 (always), adolescents rated each item on how often they had conflicts with their parents during the past 6 months. The higher the mean value of the scale is, the higher the conflict frequency is. The Cronbach's alpha for the conflict frequency scale was 0.74.

#### Adolescents' conflict coping tactics

Considering the similarity of Chinese and Japanese cultures, we used a conflict coping tactics scale developed by Japanese scholars (Ohbuchi and Tedeschi, 1997) and revised by Chinese scholars (Tu et al., 2008). The scale consists of the tactics used on mother (adolescent-mother) and father (adolescent-father) and measures four types of coping tactics: conciliation, assertion, third-party intervention and avoidance. On a 3-point Likert scale, ranging from 1 (not likely) to 3 (likely), adolescents rated respectively how they would respond to conflict with their mothers and their fathers. The scale consists of 11 items, four items for conciliation, four items for assertion, two items for third-party intervention, and one item for avoidance. The sample items include: “Persuading father/mother calmly and patiently” (conciliation), “Insisting on my position intensively” (assertion), “Asking others to help solve problems” (third-party intervention), and “Avoiding and keeping away from him/her” (avoidance). Because the sub-scales have different numbers of items, the scores were averaged across items. The higher the average, the more inclined the adolescent is to adopt certain coping tactics.

For the conflict coping tactics scale, because the sub-scale of avoidance had only one item and the reliability and validity were not reported, the current study revised the avoidance sub-scale. Before revising this sub-scale, we referenced to withdrawal sub-scale of Conflict Resolution Style Inventory (CRSI) which includes positive problem solving, conflict engagement, withdrawal, and compliance (Kurdek, 1994) and considered Chinese culture, and then added two items to the sub-scale of avoidance. The CFA was conducted to examine the factor structure of conflict coping tactics scale. The results indicated that the four factor model fitted the data adequately (for adolescent-father: *df* = 48, χ^2^ = 225.05, RMSEA = 0.04, CFI = 0.97, TLI = 0.96; for adolescent-mother: *df* = 48, χ^2^ = 223.59, RMSEA = 0.04, CFI = 0.97, TLI = 0.96). The standard pattern coefficients for the items on their respective factors ranged between 0.58 and 0.91 for adolescent-father and between 0.46 and 0.96 for adolescent-mother. According to the results of CFA, we deleted one item from the avoidance sub-scale. The revised scale consists to 12 items, four items for conciliation, four items for assertion, two items for third-party intervention, and two item for avoidance. All four sub-scales had adequate Cronbach's alphas for adolescent-father/adolescent-mother: conciliation (α = 0.79∕0.78), assertion (α = 0.72∕0.70), third-party intervention (α = 0.80∕0.79), and avoidance (α = 0.69∕0.67).

#### Life satisfaction

To measure adolescent's life satisfaction, they were asked to complete the revised version of Multidimensional Students' Life Satisfaction Scale (MSLSS, Huebner, 1994). MSLSS consists of five sub-scales: family satisfaction, self-satisfaction, friend satisfaction, school satisfaction, and living condition satisfaction. We used a 4-point Likert scale ranging from 1 (strongly disagree) to 4 (strongly agree). All five sub-scales consisted of five items. Example items included: “My family gets along well together” (family satisfaction), “I like myself” (self-satisfaction), “I have a lot of fun with my friends” (friend satisfaction), “I learn a lot at school” (school satisfaction), “I like my neighborhood” (living condition satisfaction). Higher mean value in this scale is associated with increased adolescent satisfaction with their lives. The Cronbach's alpha for the life satisfaction scale was 0.93. All five sub-scales had adequate Cronbach's alphas: family satisfaction (α = 0.88), self-satisfaction (α = 0.84), friend satisfaction (α = 0.89), school satisfaction (α = 0.87), living condition satisfaction (α = 0.82).

### Procedure

This study was approved by the Ethics Board at the School of Psychology, Beijing Normal University. Prior to the formal survey, the adolescents, and their parents were given a brief description of the study in a parents' meeting at school, and were asked to provide written informed consent. Right before the formal survey started, a research assistant read aloud the instructions and illustrated how to answer and all the questionnaires were completed in the classroom. After the students filled in the questionnaires, all papers were collected.

### Statistical analysis

Data analysis was carried out using SPSS 17.0 and AMOS 17.0. First, MANOVA was used to describe grade and gender differences in the four conflict coping tactics. To examine tactic differences of adolescents coping with their mother and their father in parent-adolescent conflicts, several paired sample *T*-tests were performed in each grade and gender. Second, Zero-order Pearson correlations were computed to assess the pair-wise relationships among conflict frequency, four conflict coping tactics and life satisfaction. Third, to examine the moderating effects of conflict coping tactics on the relationship between adolescent-parent conflicts and life satisfaction, hierarchical regression procedures were performed as recommended by Baron and Kenny (1986).

## Results

### The differences of coping tactics in adolescents' grade and gender and parents' gender

To examine the impact of adolescents' grade and gender on the four tactics coping with mother and father, a multivariate analysis of variance (MANOVA) was conducted to examine differences between the four grades (Grade 7, 8, 10, 11) and gender (boy and girl) on the eight variables (four tactics coping with mother and father). The overall MANOVA results indicated a significant overall main effect of grades (Wilks' lambda = 0.94, *F*_(24, 5392)_ = 4.91, *p* < 0.001, partial η^2^ = 0.021), gender (Wilks' lambda = 0.99, *F*_(8, 1859)_ = 2.35, *p* < 0.05, partial η^2^ = 0.010), and no significant grades × gender interaction on these variables. Table 1 presented the means, standard deviations, and MANOVA results for the research variables. For grades, the adolescents' reported using assertion and avoidance with either father or mother increased from Grade 7 to Grade 8 and remained unchanged from Grade 8 to Grade 11. For gender, boys reported using more third-party intervention to cope with father than girls did. Girls reported using more avoidance to cope with either mother or father than boys did.

**Table 1:** **Means (***M***), Standard Deviations (***SD***), and MANOVA for the study variables**.

**Variables**		**Grade 7 (*n* = 268)**	**Grade 8 (*n* = 288)**	**Grade 10 (*n* = 795)**	**Grade 11 (*n* = 523)**	**Boy (*n* = 817)**	**Girl (*n* = 1057)**	**Gender *F* partial η^2^**	**Grade *F* partial η^2^**	***Post-hoc* tests**
Conciliation	A–M	2.31(0.53)	2.31(0.55)	2.35(0.49)	2.38(0.52)	2.37(0.50)	2.32(0.52)	2.13 0.001	1.97 0.003	–
	A–F	2.26(0.54)	2.24(0.57)	2.26(0.53)	2.28(0.56)	2.28(0.54)	2.25(0.55)	1.15 0.001	0.25 0.000	–
*t* (Cohen's *d*)		2.18^*^ (0.09)	2.89^**^ (0.13)	7.64^***^ (0.18)	6.08^***^ (0.19)	7.14^***^ (0.17)	7.00^***^ (0.13)			
Assertion	A–M	1.62(0.49)	1.79(0.51)	1.80(0.48)	1.81(0.47)	1.76(0.48)	1.79(0.49)	1.14 0.001	10.48^***^ 0.017	8 = 10 = 11 > 7
	A–F	1.58(0.47)	1.75(0.53)	1.79(0.49)	1.79(0.49)	1.76(0.50)	1.75(0.50)	0.10 0.000	13.57^***^ 0.021	8 = 10 = 11 > 7
*t* (Cohen's *d*)		1.64 (0.08)	2.09^*^ (0.08)	1.08 (0.02)	1.04 (0.04)	0.05 (0.00)	3.74^***^ (0.08)			
Third-party intervention	A–M	1.53(0.60)	1.56(0.62)	1.53(0.62)	1.47(0.60)	1.54(0.61)	1.50(0.61)	1.47 0.001	1.52 0.002	8 > 11
	A–F	1.50(0.60)	1.53(0.60)	1.53(0.62)	1.46(0.59)	1.54(0.62)	1.48(0.59)	4.59^*^ 0.002	1.45 0.002	10 > 11
*t* (Cohen's *d*)		1.41 (0.05)	1.49 (0.05)	−0.23 (0.00)	1.11 (0.02)	−0.24 (0.00)	2.54^*^ (0.03)			
Avoidance	A–M	1.56(0.59)	1.79(0.64)	1.87(0.64)	1.84(0.64)	1.74(0.61)	1.86(0.66)	7.79^**^ 0.004	15.43^***^ 0.024	8 = 10 = 11 > 7
	A–F	1.58(0.60)	1.77(0.66)	1.92(0.65)	1.89(0.66)	1.80(0.64)	1.88(0.67)	2.95^*^ 0.002	20.29^***^ 0.032	8 = 10 = 11 > 7 10 = 11 > 8
*t* (Cohen's *d*)		−0.72 (−0.03)	0.49 (0.03)	−3.41^**^ (−0.08)	−2.93^*^ (−0.08)	−4.14^***^ (−0.10)	−1.53			

*M(SD). A–M, adolescents' coping tactics when they conflicted with mother; A-F, adolescents' coping tactics when they conflicted with father. The data of conflict frequency and life satisfaction do not distinguish father and mother*.

* *p < 0.05*;

** *p < 0.01*;

*** *p < 0.001*.

To examine differences in tactics that adolescents used in coping with mother and father, several paired sample *T*-tests were performed for each grade and gender. For grades, the results of Table 1 showed that adolescents adopted more conciliation in Grade 7, more conciliation and assertion in Grade 8, more conciliation and less avoidance in Grade 10 and 11 coping with mothers than with fathers. For gender, Table 1 showed that boys adopted more conciliation and less avoidance to cope with mothers than with fathers. Girls used more conciliation, assertion and third-party intervention to cope with mothers than with fathers in parent-adolescent conflicts.

In sum, as expected, there were coping tactic differences in adolescents' grade and gender and parents' gender in parent-adolescent conflicts.

### Correlations of conflict frequency, conflict coping tactics and life satisfaction

The correlations of the variables in this study are shown in Table 2. The results showed significant correlations between the study variables. Moreover, there was a highly identical correlations between below the diagonal and above the diagonal. Specifically, conflict frequency was negatively related to life satisfaction and conciliation, positively related to assertion and avoidance. Life satisfaction was positively related to conciliation and negatively related to assertion, third-party intervention and avoidance.

**Table 2:** **Inter-correlations among conflict frequency, coping tactics, and life satisfaction**.

**Variable**	**1**	**2**	**3**	**4**	**5**	**6**
1. Conflict frequency	–	−0.30^***^	0.36^***^	0.10^***^	0.29^***^	−0.29^***^
2. Conciliation	−0.29^***^	–	−0.21^***^	0.04	−0.30^***^	0.37^***^
3. Assertion	0.35^***^	−0.21^***^	–	0.24^***^	0.34^***^	−0.20^***^
4. Third-party intervention	0.12^***^	0.03	0.23^***^	–	0.10^***^	−0.07^**^
5. Avoidance	0.31^***^	−0.28^***^	0.31^***^	0.12^***^	–	−0.22^***^
6. Life satisfaction	−0.29^***^	0.35^***^	−0.20^***^	−0.07^**^	−0.26^***^	–

Correlations between coping tactics with mother and other variables are presented below the diagonal; correlations between coping tactics with father and other variables are presented above the diagonal.

** *p < 0.01*;

*** *p < 0.001*.

### Moderating effects of conflict coping tactics

To examine whether the demographic variables of adolescents' gender and grade must be used as control variables in the later regression analyses, we performed a One-way ANOVA and independent-sample *T*-Test with the two demographic variables as fixed factors and life satisfaction as the dependent variable. The results found a significant difference in gender (*t* = −2.37, *p* < 0.05). Specifically, boys scored lower than girls on life satisfaction. The differences in grade were not significant. Based on the result of these analyses, we decided to use adolescents' gender as control variables in the following regression analyses.

To examine the moderating effects of conflict coping tactics on the relationship between adolescent-parent conflicts and life satisfaction, hierarchical regression procedures were performed as recommended by Baron and Kenny (1986). Analyses were conducted respectively for adolescent-mother and adolescent-father coping tactics. Before regression analyses, predictive variables were centered by subtracting each score from the mean score of the variables. Furthermore, the present study conducted a diagnosis of multicollinearity. Chatterjee and Price (1991) proposed that variance-inflation factor (VIF) was as a diagnostic criterion of multicollinearity in regression. Generally, VIF ≥ 5 was considered multicollinear. All variance-inflation factors were below 1.30 in the present regressions, which suggested there was not multicollinearity in the present study. In the first step of each regression, gender was entered as control variables. The primary effects of conflict frequency and conflict coping tactics were entered in the second step of each regression. In the third step of each regression, the four interaction terms between conflict frequency and conflict coping tactics (conciliation, assertion, third-party intervention, and avoidance) were entered simultaneously.

A significance level of 0.01 was used due to the large sample size in the present study. Table 3 shows the standardized betas and *R*^2^-values for the subsequent steps in the regression analyses.

**Table 3:** **Hierarchical regression of satisfaction on conflict frequency and conflict coping tactics**.

**Predictor**	**Life satisfaction (*****n*** = **1874)**			
	**Adolescent-father**		**Adolescent-mother**	
	**Δ*R*^2^**	**β**	**Δ*R*^2^**	**β**
Step 1: Demographics	0.003^*^		0.003^*^	
Gender		0.06^*^		−0.09^*^
Step 2: Main effects	0.18^***^		0.18^***^	
Conflict frequency		−0.16^***^		−0.16^***^
Conciliation		0.29^***^		0.26^***^
Assertion		−0.05^*^		−0.05
Third-party intervention		−0.04		−0.03
Avoidance		−0.07^**^		−0.12^***^
Step 3: Interaction effects	0.003		0.007^**^	
Cf × Con		−0.02		−0.05^*^
Cf × Ass		−0.00		0.03
Cf × Third		−0.02		−0.02
Cf × Avo		0.04		0.05^*^
Total R2	0.18^***^		0.18^***^	

*Cf, conflict frequency; Con, conciliation; Ass, assertion; Third, third-party intervention; Avo, avoidance; adolescent-father, adolescents' coping tactics with father; adolescent-mother, adolescents' coping tactics with mother*.

* *p < 0.05*;

** *p < 0.01*;

*** *p < 0.001*.

In step 1, the gender difference in life satisfaction was statistically significant, *t* = −2.37, *p* < 0.05. Specifically, girls were more satisfied with their lives than boys.

In step 2, the primary effects of conflict frequency and coping tactics on life satisfaction were significant when gender in Step 1 was controlled. Conflict frequency had negatively predicted effects on life satisfaction (β = −0.16, *p* < 0.001). For conflict coping tactics, conciliation positively predicted life satisfaction (β = 0.29, *p* < 0.001 for adolescent-father; β = 0.26, *p* < 0.001 for adolescent-mother); avoidance negatively predicted life satisfaction (β = −0.07, *p* < 0.01 for adolescent-father; β = −0.12, *p* < 0.001 for adolescent-mother). These results support the hypothesis in the present study.

In step 3, although the moderating effects were significant at the level of 0.05, Step 3 explained less than 1% of the variance. Due to the large sample size, the present study adopted a stricter *p* < 0.01). Adopting the stricter standard, the moderating effects were all not significant, which were not in line with the hypothesis in the present study.

## Discussion

This cross-sectional study examined the difference of conflict coping tactics in adolescents' grade and gender and parents' gender in parent-adolescent conflicts and explored the relationships among conflict frequency, conflict coping tactics, and life satisfaction among 1874 adolescents in China. The results showed that there were differences of conflict coping tactics in adolescents' grade and gender and parents' gender, and conflict frequency and coping tactics played different roles in predicting adolescents' life satisfaction.

### The differences of coping tactics in adolescents' grade and gender and parents' gender

As expected, there were grade and gender differences and parent gender differences in conflict coping tactics in parent-adolescent conflicts.

First, adolescents' coping tactics with mother and father changed with grade. The use of assertion and avoidance increased with grade. These findings are inconsistent with previous results which showed that adolescents' reported use of conflict engagement and avoidance with mothers first temporarily increased and then decreased (Van Doorn et al., 2011). The cause of these inconsistent findings can be explained by the cultural difference between Western and Chinese cultures. Today, Chinese adolescents no longer entirely obey their parents as traditionally they would be. They have become more independent from parents and value freedom and autonomy significantly more (Li et al., 2014). Thus, adolescents begin to be more assertive when parent-adolescent conflicts occur, resulting in the increase of reported use of assertion with grade. However, Chinese adolescents cannot completely break away from the influence of a collectivist culture which emphasizes interpersonal connectedness much more than individuality (Markus and Kitayama, 1991). As a result their reported use of avoidance increased with grade. Conciliation scored highest among all tactics at all grade levels. Conciliation did not increase with grade, but the higher score implies that more mature conflict resolution were developed during adolescence (Sandy and Cochran, 2000). In addition, Chinese adolescents used less third-party intervention overall. It is worth noting that from whom adolescents would seek support during parent-adolescent conflicts: father, mother, siblings, or others. Due to the Chinese one-child policy during the past several decades, adolescents tend to be overprotected by their parents. Compared with adolescents in Western countries, they have no chance to learn problem-solving skills from interactions with siblings. Moreover, the traditional Chinese mentality is that any family disharmony should be kept within the family. It is a shame to reveal family problems to outsiders. For this cultural reason, there are relatively few institutions/organizations in China, such as unions and the judicial system, to function as third-party intervention forces. Thus, adolescents may lack the awareness of seeking help from a third party when facing conflicts.

Second, differences in adolescents' tactics for coping with mothers and with fathers were significant in each grade. The results indicated that adolescents adopted more conciliation in Grade 7, more conciliation and assertion in Grade 8, more conciliation and less avoidance in Grade 10 and 11 in coping with mothers than with fathers. There is an old saying in China, “Man goes out to work while woman looks after the house” (Chinese: Nan Zhu Wai, Nyu Zhu Nei). Due to the differences in parents' gender role in Chinese culture, mothers spend more time in care-giving, joint activities, and conversation with adolescents than fathers do (Lewis and Lamb, 2003; Parke and Buriel, 2006). Some studies have also shown that adolescents described themselves as closer to mothers than to fathers (Youniss and Smollar, 1985; Cubis et al., 1989). Therefore, adolescents adopted more direct and active tactics (e.g., conciliation and assertion) to cope with mothers than with fathers, and adopted more indirect and passive tactics (e.g., avoidance) to cope with fathers than with mothers. In addition, the influence of parents' authority on adolescents is also far-reaching, especially the father's authority. There are still the remnants of the traditional concept of hierarchy in today's Chinese mentality. Because of the belief of submitting to the authority of the father, adolescents tend to use more avoidance coping tactics with the father than with the mother.

Third, as for gender, girls adopted more avoidance to cope with mothers and fathers than boys did as a whole. This result is consistent with previous findings which indicated that girls report greater use of avoidance than boys (Owens et al., 2005). Moreover, our results from this study indicated boys adopted more conciliation and less avoidance to cope with mothers than with fathers, girls adopted more conciliation, assertion, and third-party intervention to cope with mothers than with fathers. The previous research indicated that mothers tended to compromise more than fathers (García-Ruiz et al., 2013). Gender differences exist in the findings that boys adopted more avoidance tactics to cope with fathers than with mothers, while girls adopted more assertion and third-party intervention tactics to cope with mothers than with fathers in parent-adolescent conflicts. The results are partially in line with the previous research findings, which indicated that the female participants used third-party intervention relatively more strongly than did the male participants (Ohbuchi et al., 1999). Again this may be caused by the cultural difference. For example, adolescents use more avoidance and less assertion in the collectivist culture (Ohbuchi et al., 1999). Especially in Chinese culture, father is more strict with son than with daughter. The old Chinese saying goes, “Boys should be raised in poverty, girls in wealth” (Chinese: Qiong Yang Er, Fu Yang Nyu). Thus, boys used more avoidance tactics to cope with father than mother.

### The relationships among conflict frequency, coping tactics, and life satisfaction

As expected, there was a primary effect of conflict frequency and conflict coping tactics on adolescents' life satisfaction. We found that life satisfaction was negatively predicted by conflict frequency and avoidance (ineffective tactic) and was positively predicted by conciliation (effective tactic) after controlling adolescents' gender. These findings are consistent with the existing findings. Existing studies have demonstrated that effective resolutions were beneficial to adolescents' well-being and adjustment (Tucker et al., 2003), and ineffective resolutions led to detrimental consequences (Van Doorn et al., 2008). However, no associations were found between third-party intervention and life satisfaction and between assertion and life satisfaction when we adopted a stricter *p* < 0.01). Overall, adolescents used less third-party intervention to solve the conflicts with their parents. Moreover, adolescents' life satisfaction were influenced by many factors, such as maternal concern (parenting style) (Leung et al., 2004), family functioning (Shek, 2002), and self-esteem (Tucker et al., 2003). Therefore, life satisfaction was mixed with other factors, such as family relationships and self-esteem, which could lead to a decrease in the predictive effect. If possible, further studies can explore more specific outcomes.

In addition, inconsistent with hypothesis, the results indicated that the moderating effects were not significant when the present study adopted a stricter *p* < 0.01). The possible reason is that adolescents will use combined rather than isolated strategy to cope with conflicts with their parents. For example, using the hierarchical cluster analysis, Branje et al. (2009) distinguished five conflict resolution styles: withdraw, positive, negative, no resolution, and very positive. And the same strategy, as an isolated strategy or a combination of strategies, could play different roles in the relationship between conflict frequency and adolescents' life satisfaction. Hence, combining different strategies into a proper resolution and examining their effects on life satisfaction are the next step for this study.

### Limitations and future directions

There are three limitations in this study. First, it is a cross-sectional study, therefore, the causal relationships cannot be truly established. Future studies are needed to reconfirm these findings. Also, a longitudinal study is needed to examine the developmental trend of coping tactics with mothers and with fathers. Second, the study only included one informant (adolescents) in parent-adolescent conflicts. Perceived adolescent-parent relationships are different for different family members (Sillars et al., 2010). For example, a longitudinal study on developmental changes in conflict resolution styles in parent-adolescent relationships showed that adolescents' reported positive problem-solving with mothers increased, but had no change with fathers. Fathers reported an increase of positive problem solving with adolescents, whereas mothers reported no change (Van Doorn et al., 2011). Future research may consider different family members in assessing conflict frequency and conflict coping tactics. Third, conflict frequency is assessed without separating mother and father. The frequency and contents of conflicts with father and with mother are different (Fang et al., 2003). Although this study mainly focused on the differences of coping tactics, rather than the differences of conflict frequency, in parents' gender, mother-adolescent conflicts may be different from father-adolescent conflicts, which may have a different effect on life satisfaction. Future research may consider the assessment of conflict frequency for mother and father separately in order to accurately examine the relationships among conflict frequency, coping tactics and life satisfaction. Finally, the use of self-report as an assessment of conflict coping tactics, other than the use of real conflict situation or recalling of real parent-adolescent conflict-resolving process, which does not assure ecological validity of the research and collects richer firsthand information (Burk et al., 2009; Sillars et al., 2010). Hence, exploration of the relationships among conflict, conflict coping tactics and life satisfaction in real conflict-resolving situation is needed.

### Implications

The current study has the following two primary contributions. First, the study revealed the differences of coping tactics in terms of adolescents' grade and gender and parents' gender in the context of Chinese culture. Second, this study analyzed the relationships among conflict frequency, coping tactics, and adolescents' life satisfaction. These results expanded and supplemented the existing cross-cultural studies of the relationship between parent-adolescent conflicts and life satisfaction. And these findings also provided a new perspective for the future research on conflict and well-being. In addition, clinicians and psychological counselors should take these differences of adolescents' conflict coping tactics with mother and with father into considerations in their practices.

## Conclusions

The present study examined the differences of conflict coping tactics in adolescents' grade and gender and parents' gender in parent-adolescent conflicts, and explored the relationships among conflict frequency, conflict coping tactics, and life satisfaction. The results indicated: first, there were significant differences of coping tactics in adolescents' grade, gender, and parents' gender. Second, the main effects of conflict frequency and conflict coping tactics on adolescents' life satisfaction were significant. Third, there was no significant moderating effects of coping tactics with mother and with father.

### Conflict of interest statement

The authors declare that the research was conducted in the absence of any commercial or financial relationships that could be construed as a potential conflict of interest.

## References

[B1] Adams R. Laursen B. (2001). The organization and dynamics of adolescent conflict with parents and friends. J. Marr. Fam. 63, 97–11. 10.1111/j.1741-3737.2001.00097.x

[B2] Adams R. Laursen B. (2007). The correlates of conflict: disagreement is not necessarily detrimental. J. Fam. Psychol. 21, 445–458. 10.1037/0893-3200.21.3.445 17874930 PMC2422875

[B3] Baron R. M. Kenny D. A. (1986). The moderator–mediator variable distinction in social psychological research: conceptual, strategic, and statistical considerations. J. Pers. Soc. Psychol. 51, 1173–1182. 10.1037/0022-3514.51.6.1173 3806354

[B4] Bem S. L. (1985). Androgyny and gender schema theory: a conceptual and empirical integration, in Nebraska Symposium on Motivation, Vol. 32 (Lincoln, OR: University of Nebraska Press), 179–226. 6398855

[B5] Bosma H. A. Kunnen E. S. (2001). Determinants and mechanisms in ego identity development: a review and synthesis. Dev. Rev. 21, 39–66. 10.1006/drev.2000.0514

[B6] Bradley R. H. Corwyn R. F. (2004). Life satisfaction among European American, African American, Chinese American, Mexican American, and Dominican American adolescents. Int. J. Behav. Dev. 28, 385–400. 10.1080/01650250444000072

[B7] Branje S. J. T. Van Doorn M. Valk I. V. D. Meeus W. (2009). Parent-adolescent conflicts, conflict resolution types, and adolescent adjustment. J. Appl. Dev. Psychol. 30, 195–204. 10.1016/j.appdev.2008.12.004

[B8] Burk W. J. Denissen J. Van Doorn M. D. Branje S. J. T. Laursen B. (2009). The vicissitudes of conflict measurement: stability and reliability in the frequency of disagreements. Eur. Psychol. 14, 153–159. 10.1027/1016-9040.14.2.153

[B9] Chatterjee S. Price B. (1991). Regression Analysis by Example. New York, NY: John Wiley and Sons.

[B10] Cicognani E. Zani B. (2010). Conflict styles and outcomes in families with adolescent children. Soc. Dev. 19, 427–437. 10.1111/j.1467-9507.2009.00545.x

[B11] Collins W. A. Laursen B. (1992). Conflicts and relationships during adolescence, in Conflict in Child and Adolescent Development, eds Shantz C. U. Hartup W. W. (New York, NY: Cambridge University Press), 216–241.

[B12] Colsman M. Wulfert E. (2002). Conflict resolution style as an indicator of adolescents substance use and other problem behaviors. Addict. Behav. 27, 633–648. 10.1016/S0306-4603(01)00198-8 12188597

[B13] Cooper C. R. (1999). Multiple selves, multiple worlds: cultural perspectives on individuality and connectedness in adolescent development, in Cultural Processes in Child Development: The Minnesota Symposia on Child Psychology, Vol. 29, eds Masten A. S. (Mahwah, NJ: Erlbaum), 25–57.

[B14] Cubis J. Lewin T. Dawes F. (1989). Australian adolescents' perceptions of their parents. Aust. N. Z. J. Psychol. 23, 35–47. 10.3109/00048678909062590 2930414

[B15] Deković M. (1999). Parent-adolescent conflict: possible determinants and consequences. Int. J. Behav. Dev. 23, 977–1000. 10.1080/016502599383630

[B16] Fang X. Y. Zhang J. T. Liu Z. (2003). Parent-adolescent conflict. Psychol. Dev. Educ. 3, 46–52. 14665739

[B17] Feldman S. S. Fisher L. Ransom D. C. Dimiceli S. (1995). Is “what is good for the goose good for the gander?” Sex differences in relations between adolescent coping and adult adaptation. J. Res. Adolesc. 5, 333–359. 10.1207/s15327795jra0503_3

[B18] Frydenberg E. Lewis R. (1991). Adolescent coping: the different ways in which boys and girls cope. J. Adolesc. 14, 119–133. 10.1016/0140-1971(91)90025-M 1918514

[B19] García-Ruiz M. Rodrigo M. J. Hernández-Cabrera J. A. Máiquez M. L. (2013). Contribution of parents' adult attachment and separation attitudes to parent-adolescent conflict resolution. Scand. J. Psychol. 54, 459–467. 10.1111/sjop.12077 24117437

[B20] Gil-Rivas V. Greenberger E. Chen C. Lopez-Lena M. M. (2003). Understanding depressed mood in the context of a family-oriented culture. Adolesc. 38, 93–109. 12803456

[B21] Henry C. S. (1994). Family system characteristics, parental behaviors, and adolescent family life satisfaction. Fam. Relatsh. 43, 447–455. 10.2307/585377

[B22] Ho D. Y. F. Chen S. J. Chiu C. Y. (1991). Relational orientation: in search of methodology for Chinese social psychology, in Chinese Psychology and Behavior, eds Yang K. S. Hwang K. K. (Taipei: Laureate), 49–66.

[B23] Huebner E. S. (1994). Preliminary development and validation of a multidimensional life satisfaction scale for children. Psychol. Assess. 6, 149–158. 10.1037/1040-3590.6.2.149

[B24] Huebner E. S. Drane W. Valois R. F. (2000). Levels and demographic correlates of adolescent life satisfaction reports. Sch. Psychol. Int. 21, 281–292. 10.1177/0143034300213005

[B25] Hwang K. K. (2000). Chinese relationalism: theoretical construction and methodological considerations. J. Theory Soc. Behav. 30, 155–178. 10.1111/1468-5914.00124

[B26] Juang L. P. Syed M. Cookston J. T. Wang Y. Kim S. Y. (2012). Acculturation-based and everyday family conflict in Chinese American Families. New Dir. Child Adoles. 135, 13–34. 10.1002/cd.20002 PMC4777626 22407880

[B27] Kurdek L. A. (1994). Conflict resolution styles in gay, lesbian, heterosexual nonparent, and heterosexual parent couples. J. Marr. Fam. 56, 705–722. 10.2307/352880

[B28] Lam C. M. (1997). A cultural perspective on the study of Chinese adolescent development. Child Adolesc. Soc. Work J. 14, 95–113. 10.1023/A:1024553132465

[B29] Laursen B. Coy K. C. Collins W. A. (1998). Reconsidering changes in parent-child conflict across adolescence: a meta-analysis. Child Dev. 69, 817–832. 10.1111/j.1467-8624.1998.tb06245.x 9680687 PMC2730212

[B30] Laursen B. Hafen C. A. (2010). Future directions in the study of close relationships: conflict is bad (expect when it's not). Soc. Dev. 19, 858–872. 10.1111/j.1467-9507.2009.00546.x 20953335 PMC2953261

[B31] Leung C. Y. W. McBride-Chang C. Lai B. P. Y. (2004). Relations among maternal parenting style, academic competence, and life satisfaction in Chinese early adolescents. J. Early Adolesc. 24, 113–143. 10.1177/0272431603262678

[B32] Leung J. Leung K. (1992). Life satisfaction, self-concept, and relationship with parents in adolescence. J. Youth Adolesc. 21, 653–665. 10.1007/BF01538737 24264168

[B33] Lewis C. Lamb M. E. (2003). Fathers' influences on children's development: the evidence from two-parent families. Eur. J. Psychol. Educ. 18(2): 211–228. 10.1007/BF03173485

[B34] Li X. W. Zou H. Liu Y. Zhou Q. (2014). The relationships of family socioeconomic status, parent-adolescent conflict, and filial piety to adolescents' family functioning in mainland China. J. Child Fam. Stud. 23, 29–38. 10.1007/s10826-012-9683-0

[B35] Markus H. R. Kitayama S. (1991). Culture and the self: implications for cognition, emotion, and motivation. Psychol. Rev. 98, 224–253. 10.1037/0033-295X.98.2.224

[B36] McCubbin H. I. (1979). Integrating coping behavior in family stress theory. J. Marr. Fam. 41, 237–244. 10.2307/351693

[B37] Montemayor R. (1983). Parents and adolescents in conflict: all families some of the time and some families most of the time. J. Early Adolesc. 3, 83–103.

[B38] Ohbuchi K. I. Chiba S. Fukushima O. (1996). Mitigation of interpersonal conflicts: politeness and time pressure. Pers. Soc. Psychol. Bull. 22, 1035–1042. 10.1177/01461672962210007

[B39] Ohbuchi K. I. Fukushima O. Tedeschi J. T. (1999). Cultural values in conflict management: goal orientation, goal attainment, and tactical decision. J. Cross Cult. Psychol. 30, 51–71. 10.1177/0022022199030001003

[B40] Ohbuchi K. I. Takahashi Y. (1994). Cultural styles of conflict management in Japanese and American: passivity, covertness, and effectiveness of strategies. J. Appl. Soc. Psychol. 24, 1345–1366. 10.1111/j.1559-1816.1994.tb01553.x

[B41] Ohbuchi K. Tedeschi J. T. (1997). Multiple goals and tactical behavior in social conflicts. J. Appl. Soc. Psychol. 27, 2177–2199. 10.1111/j.1559-1816.1997.tb01647.x

[B42] Osterman K. Bjorkqvist K. Lagerspetz K. M. J. Landau S. F. Fraczek A. Pastorelli C. (1997). Sex differences in styles of conflict resolution: a developmental and cross-cultural study with data from Finland, Israel, Italy, and Poland, in Cultural Variation in Conflict Resolution: Alternatives to Violence, eds Fry D. P. Bjorkqvist K. (Mahwah, NJ: Lawrence Erlbaum), 185–197.

[B43] Owens L. Daly A. Slee P. (2005). Sex and age differences in victimization and conflict resolution among adolescents in a South Australian school. Aggr. Behav. 31, 1–12. 10.1002/ab.20045

[B44] Parke R. D. Buriel R. (2006). Socialization in the family: ethnic and ecological perspectives, in The Handbook of Child Psychology. Social, Emotional, and Personality Development, 6th Edn., Vol. 3, eds Damon W. Lerner R. M. Eisenberg N. (New York, NY: Wiley), 429–504.

[B45] Pruitt D. G. Carnevale P. J. (1993). Negotiation in Social Conflict. Pacific Grove, CA: Thomson Brooks/Cole Publishing Co.

[B46] Russell A. Saebel J. (1997). Mother–son, mother–daughter, father–son, and father–daughter: are they distinct relationships? Dev. Rev. 17, 111–147. 10.1006/drev.1996.0431

[B47] Sandy S. V. Cochran K. (2000). The development of conflict resolution skills in children: Preschool to adolescence, in The Handbook of Conflict Resolution: Theory and Practice, eds Deutsch M. Coleman P. (San Francisco, CA: Jossey-Bass), 316–342.

[B48] Shantz C. U. (1987). Conflict between children. Child Dev. 58, 283–305. 10.2307/1130507

[B49] Shek D. T. L. (2002). Family functioning and psychological well-being, school adjustment, and problem behavior in Chinese adolescents with and without economic disadvantage. J. Gene Psychol. 163, 497–502. 10.1080/00221320209598698 12495233

[B50] Sillars A. Smith T. Koerner A. (2010). Misattributions contributing to empathic (in) accuracy during parent-adolescent conflict discussions. J. Soc. Pers. Relatsh. 27, 727–747. 10.1177/0265407510373261

[B51] Silverberg S. B. Steinberg L. (1987). Adolescent autonomy, parent-adolescent conflict, and parental well-being. J. Youth Adolesc. 16, 293–312. 10.1007/BF02139096 24277374

[B52] Smetana J. Gaines C. (1999). Adolescent-parent conflict in middle-class African American families. Child Dev. 70, 1447–1463. 10.1111/1467-8624.00105 10621966

[B53] Smetana J. G. (1996). Adolescent-parent conflict: implications for adaptive and maladaptive development, in Rochester Symposium on Developmental Psychopathology: Adolescence: Opportunities and Challenges, eds Cicchettiand D. Toth S. L. (Rochester, NY: University of Rochester), 1–46.

[B54] Steinberg L. (1990). Autonomy, conflict, and harmony in the family relationship, in At the Threshold: The Developing Adolescent, eds Feldman S. S. Elliot G. R. (Cambridge, MA: Harvard University Press), 255–276.

[B55] Thompson L. Walker A. J. (1989). Gender in families: women and men in marriage, work, and parenthood. J. Marr. Fam. 51, 845–871. 10.2307/353201

[B56] Tu C. P. Fang X. Y. Liu Z. (2008). The relationship between family environment type and parent-adolescent conflict coping. Stud. Psychol. Behav. 6, 187–191. Available online at: http://journal.psytj.net/CN/Y2008/V6/I3/187

[B57] Tucker C. J. McHale S. M. Crouter A. C. (2003). Conflict resolution links with adolescents' family relationships and individual well-being. J. Fam. Issues 24, 715–736. 10.1177/0192513X03251181

[B58] Van Doorn M. D. Branje S. J. T. Meeus W. H. J. (2008). Conflict resolution in parent-adolescent relationships and adolescent delinquency. J. Early Adolesc. 28, 503–527. 10.1177/0272431608317608

[B59] Van Doorn M. D. Branje S. J. T. Meeus W. H. J. (2011). Developmental changes in conflict resolution styles in parent-adolescent relationships: a four-wave longitudinal study. J. Youth Adolesc. 40, 97–107. 10.1007/s10964-010-9516-7 20177961 PMC3003790

[B60] West C. Zimmerman D. H. (1987). Doing gender. Gend. Soc. 1, 125–151. 10.1177/0891243287001002002

[B61] Youniss J. Smollar J. (1985). Adolescents' Relationships with Mothers, Fathers and Friends. Chicago, IL: University of Chicage Press.

